# Supplementary material for: Effects of systemic inflammation on the network oscillation in the anterior cingulate cortex and cognitive behavior
Source: PLoS One. 2024 May 3;19(5):e0302470. doi: 10.1371/journal.pone.0302470 (PMC11068183; doi:10.1371/journal.pone.0302470)
Supplement: Attachment — Submitted filename: 240329 AnswersToReviewer.docx [file pone.0302470.s004.docx]

Dear Editors and Reviewers,

Thank you very much for reviewing our manuscript and giving us invaluable comments.

Please find our point-by-point responses in the following pages.

We reanalyzed our data following the reviewer 2’s comments. As a consequence, Table 1 and Fig 6B and C were replaced with the new ones, and original Table 1 is presented as Supplemental Table 1.

Conclusions of the manuscript is preserved after the reanalysis, and several changes were made in the text to meet the suggestions by the reviewer 2.

**Reviewer #2: I strongly encourage to tone down the statements involving causal involvement of ACC oscillations in behavioural readouts throughout the manuscript. This is also true for the involvement of Histamine as a mediator of the reported effects. First, there is only a minor correlation with a behavioural parameter in the open field test. Second, the oscillations recorded in this study are ex vivo/in vitro and their causal involvement in behavioural readouts are of correlative nature without any causal evidence at this stage.**

Ans.)

We made changes in the manuscript following the suggestion by the reviewer to tone down the statements regarding causality or correlation. The revised sentence in the abstract, ll. 32-36, reads: These results suggest that LPS-induced systemic inflammation results in increased network oscillation and a drastic change in histamine sensitivity in the ACC, accompanied by the robust production of systemic pro-inflammatory cytokines in the periphery, and that these alterations in the network oscillation and animal behavior as an acute phase reaction relate with each other. We add a sentence in the discussion, ll.371-372, that reads: Causal relationship between network oscillation and behavior would be clarified by further studies.

**Q1
-Page 6, Line 84-85: rephrase this sentence as these are two different tasks assessing anxiety/activity and memory, respectively:
”…open field and novel object recognition, a behavioral task 84 related to ACC function (2,6)….”**

Ans. 1)

We revised the sentence as suggested by the reviewer. The revised sentence reads: We analyzed the effects of acute LPS administration on 1) kainic acid (KA)-induced network oscillation from the Cg1 region of the ACC, either in the presence or absence of histamine; 2) anxiety by open field (6); 3) novelty detection by novel object recognition test (2); and 4) interleukin (IL)-6 concentration in the serum and cerebral cortex as a surrogate marker for inflammation.

**Q2**
**-Page 8, Line137-139 and Page 18, Line314-318: It is not clear to me how the parameters assessed in the open field test are related to motivation.**

Ans. 2)

We removed the word, motivation, from the indicated sentence adopting the point raised by the reviewer.

**Q3**
**-I suggest dividing the open field test data into 5 min bins as the anxiety/activity measures are more reliable at the beginning of this test.**

Ans. 3)

We made a new table by analyzing the first 5-min data of the open field test following the reviewer’s comments. The new table (for the first 5-min) replaced the old one (for entire 10-min) in the text; the “10-min” table is presented as the S1 table. Figure panels for 6B and C also replaced ones for the first 5-min. Relevant correlations were essentially conserved for the first 5-min window analysis, with a couple of additional statistical significances. We carried out the third set of analysis for the second 5-min window data to find above mentioned significant correlations were limited to the first 5-min.

**Q4
-Regarding the correlation between the behavioural parameters and oscillation power. It would be interesting to do correlation separately within control and LPS group as the direction of the correlation can change in control vs LPS.**

Ans. 4)

We did a set of new analysis after separating control- and LPS-mice following the suggestion by the reviewer. Indeed, we noted a statistical significance in a data subset, i.e. theta band oscillatory power v.s. spent time in the center area, specifically for the control-group, which was not evident in the combined data set. A further look reveled that one or two data points in the control-group had far stronger statistical influence over the remaining ones, raising the possibility of a false-positive case; please find the attached scattergram. We concluded that it would be safer not to separate control- and LPS-groups for the current data set, considering the limited number of animals used in this study.

**Q5
-For the correlation, the time spent in the centre is not reported. I think this is a better readout then the entries. Also, the correlation should be performed with the data corresponding to the first 5 min of the open field as mentioned above.**

Ans. 5)

The time spent in the center has been reported in the center part of the Table 1. We admit the column titles lacked clarity. We modified the column titles “total entries” and “total time” to “total entries to the center area” and “total time spent in the center area”.

**Q6
There are still some minor grammatical issues that can be corrected during the proofreading stage.**

Ans. 6)

We are sorry for the grammatical errors remaining in the text. We carried out an extra round of checking to get rid of the errors as much as possible.
